# Squalene Found in Alpine Grassland Soils under a Harsh Environment in the Tibetan Plateau, China

**DOI:** 10.3390/biom8040154

**Published:** 2018-11-20

**Authors:** Xuyang Lu, Shuqin Ma, Youchao Chen, Degyi Yangzom, Hongmao Jiang

**Affiliations:** 1 Institute of Mountain Hazards and Environment, Chinese Academy of Sciences, Chengdu 610041, China; xylu@imde.ac.cn (X.L.); jianghongmao@163.com (H.J.); 2 Key Laboratory of Mountain Surface Processes and Ecological Regulation, Chinese Academy of Sciences, Chengdu 610041, China; 3 College of Tourism, Henan Normal University, Xinxiang 453007, China; 4 Wuhan Botanical Garden, Chinese Academy of Sciences, Wuhan 430074, China; chenyouchao@wbgcas.cn; 5 Ecological Monitoring & Research Center, Tibetan Environment Monitoring Station, Lhasa 850000, China; dejiyangzong09@126.com; 6 University of Chinese Academy of Sciences, Beijing 100049, China

**Keywords:** squalene, alpine grassland, Py-GC/MS, soil microorganism, Tibetan Plateau

## Abstract

Squalene is found in a large number of plants, animals, and microorganisms, as well as other sources, playing an important role as an intermediate in sterol biosynthesis. It is used widely in the food, cosmetics, and medicine industries because of its antioxidant, antistatic, and anti-carcinogenic properties. A higher natural squalene component of lipids is usually reported as being isolated to organisms living in harsh environments. In the Tibetan Plateau, which is characterized by high altitude, strong solar radiation, drought, low temperatures, and thin air, the squalene component was identified in five alpine grasslands soils using the pyrolysis gas chromatography–mass spectrometry (Py-GC/MS) technique. The relative abundance of squalene ranged from 0.93% to 10.66% in soils from the five alpine grasslands, with the highest value found in alpine desert and the lowest in alpine meadow. Furthermore, the relative abundance of squalene in alpine grassland soils was significantly negatively associated with soil chemical/microbial characteristics. These results indicate that the extreme environmental conditions of the Tibetan Plateau may stimulate the microbial biosynthesis of squalene, and the harsher the environment, the higher the relative abundance of soil squalene.

## 1. Introduction

Squalene is named after the shark family Squalidae, and is a triterpene with the formula C_30_H_50_. It is an intermediate in the biosynthesis of sterols and hopanoids in the plant, animal, human, and microorganism worlds [1,2]. From the moment life appeared on Earth, squalene appeared in microorganisms. The cell membranes of higher organisms from the Precambrian contained great proportions of squalene, which was an essential substance for their survival in the hostile oxygen-free environment [2]. Squalene and its related compounds such as oxidosqualene and bis-oxidosqualene are precursors of thousands of bioactive triterpenoids and are also a carbon source which can be utilized by some microorganisms [3].

Squalene itself has several beneficial properties and values. For instance, it is a hydrophilic natural antioxidant which serves in health-promoting functions including skin hydration and tumor-suppression. It has cardio-protective, antibacterial/antifungal, immunity-boosting, and cholesterol-lowering effects. It can also be used as a drug delivery agent, and has been used as a feasible source of biofuels [4,5]. Thus, squalene has recently attracted a great deal of attention due to its industrial value as a lubricant, health-promoting agent, and/or as a form of biofuel.

The richest known source of squalene in the living world is the liver of certain species of fish, especially sharks living in the deep sea [1,6]. As the main organ for lipid storage, as an energy source, and for adjusting buoyancy, the liver of sharks comprises 50–80% unsaponifiable matter, with the great majority thereof being squalene. For example, *Centrophorus artomarginatus* deep-sea sharks can survive in waters with a depth of 600–1000 m, where with the environmental characteristics include lack of sunlight, consistently high pressure, and very poor oxygen supply. This survival is due to squalene from their liver, which accounts for 25–30% of their total body weight [2,7].

Squalene was also identified in many plant oils over broad ranges. The first vegetable oil in which it was found was olive oil, with a concentration of 5.64–5.99 g kg^−1^. In other vegetable oils, it is also quite prominent in soybean, grape seed, hazelnuts, peanuts, corn, pumpkin, rice bran, amaranth, and camellia oils [2,8]. Human serum also contains 10–13% squalene as one of its major constituents [3,9]. In addition, microbial squalene production has become a promising alternative in recent years due to the advantages of fast and massive growth, although microorganisms do not accumulate as much squalene as plants or shark livers [10,11,12].

The Tibetan Plateau, as the roof of the world, is considered to be the third “pole” of the world. The plateau is peculiarly cold due to its latitude, and is colder than anywhere else outside the polar regions. It has an average elevation of 4 km above sea level, and possesses one of the largest ice masses on Earth [13,14,15]. The plants, animals, and microorganisms living in the Tibetan Plateau endure extreme circumstances, characterized by high altitude, strong solar radiation, drought, low temperatures, thin air, and so on [16,17,18]. Low temperature and low oxygen pose key physiological challenges for those living in these harsh conditions on the plateau, a situation which is to some extent similar to that of sharks living within a deep-sea environment.

Squalene has been identified from some Tibetan plant components, including the lipophilic extracts from flowers (0.29–0.77%) and leaves (0.56–1.16%) of *Lamiophlomis rotate*, and the volatile oil from roots (1.73%) of *Rhodiola crenulata* [19,20]. The Tibetan yak can thrive well at altitudes of 2000–5000 m above sea level, and provides meat, milk, and other necessities for the local people. The highest squalene content in lipids was reported to exist in the longissimus muscle (20.99 mg/100 g), the biceps femoris muscle (59.82 mg/100 g), the liver (6.94 mg/100 g), the subcutaneous adipose tissue (7.06 mg/100 g), and the abdominal adipose tissue (7.06 mg/100 g) of the Tibetan yak [21]. Therefore, the chemical component of squalene has been found in some plants and animals on the Tibetan Plateau. Could this component also be identified from some microorganisms which likewise live in high-altitude, low-temperature, low-oxygen alpine conditions? As a variety of microorganisms were found to distributed in alpine soils of the Tibetan Plateau [22,23,24], in the present study alpine soils from five types of alpine grassland were analyzed by using pyrolysis gas chromatography–mass spectrometry (Py-GC/MS) to identify the squalene component. The aim of this study was to compare squalene content among different alpine grassland soils and further to explore the relationships between the squalene content and the soil environmental factors in the harsh conditions on the Tibetan Plateau.

## 2. Materials and Methods

### 2.1. Study Area

Tibet covers a total area of more than 1.2 million km^2^ and represents is approximately one-eighth of the total area of China, with an average altitude higher than 4000 m. It regulates climate change and water resources in China and eastern Asia due to its geomorphological uniqueness in the world [25,26]. Because of its extensive territory and highly dissected topography, this region has a diverse range of climate and vegetation zones. Solar radiation is strong, with annual radiation varying between 140 and 190 kcal cm^−2^. Due to the geographical conditions and atmospheric circulation, the average annual temperature is rather low, with the temperature varying from 18 to −4 °C, decreasing gradually from the southeast to the northwest. The average annual precipitation is less than 1000 mm in most areas of Tibet; annual precipitation rates can reach up to 2817 mm in the east and drop down to approximately 70 mm in the west [27].

Alpine grasslands are the most dominant ecosystems in Tibet, covering more than 70% of the whole plateau’s area. It ranks first among all Chinese provinces and autonomous regions in the diversity of its grassland ecosystems, comprising 17 types of grassland based on the classification system used for the whole country [28,29]. Among all grassland types, alpine meadow (AM) is composed of perennial mesic and mesoxeric herbs under cold and wet climate conditions, occupying approximately 31.3% of the total grassland area of Tibet. Alpine steppe (AS) is composed of drought-tolerant perennial herbs or small shrubs under cold and arid/semiarid climate conditions, representing approximately 38.9% of the total Tibetan grassland area. Alpine desert (AD) is a grassland type developed and controlled by cold and extreme drought conditions, covering 6.71% of the total grassland area. Alpine meadow steppe (AMS) is a transitional type of alpine grassland from the meadow to the steppe, and alpine desert steppe (ADS) is a transitional type of alpine grassland from the steppe to the desert, covering 7.32% and 10.7% of the total grassland area in Tibet, respectively [27].

### 2.2. Soil Sampling

In this research, the study area was located at 30.75°–33.43° N, 79.75°–92.07° E, and the sampling sites were located in 10 counties from east to west in the Tibet Autonomous Region of China. Five sampling sites were selected at each of the three main natural grassland types, including AM, AS, and ADS. Three sampling sites were selected from the relatively small natural grassland area, including AMS and AD, in August 2016. At each sample site, three 1 m × 1 m quadrats were laid out at intervals of approximately 50 m. In total, 63 quadrats of alpine grassland in Tibet were sampled with 45 quadrats (15 sites × 3 quadrats) for AM, AS, and ADS and 18 quadrats (6 sites × 3 quadrats) for AMS and AD, respectively. At each quadrat, all aboveground plants and litter were removed from the soil surface before the sampling. Five soil samples were obtained for each quadrat at depths of 0–15 cm, and five soil samples were mixed as a soil sample for the soil chemical and microbial properties analysis. All soils were transported to the lab with cooler, and stored in sealed containers at 4 °C before the measurement. For the determination of soil bulk density, soil cores (5.4 cm in diameter) were also taken from each layer using a stainless-steel cylinder. In addition, the location and elevation of each site were measured using Global Positioning System (GPS) (Garmin MAP62CSX made in Garmin Ltd., Olathe, KS, USA).

### 2.3. Soil Analyses

In the lab, soil samples for soil chemical and microbial properties analyses were sieved to pass through a 2-mm-mesh sieve, and roots and stones were removed by hand. Then the samples were divided into three sub-samples. One sub-sample was air-dried and the squalene component was identified by pyrolysis gas chromatography–mass spectrometry (Py-GC/MS); the second was stored at 4 °C prior to determine soil microbial phospholipid fatty acids (PLFAs); and the third was sieved through 250-μm mesh for analysis of soil pH, soil organic carbon (SOC), dissolved organic carbon (DOC), total nitrogen (TN), and total inorganic nitrogen (TIN) contents.

Py-GC/MS tests were performed in a pyrolyzer (CDS5200). For this, 25 mg of soil was placed in quartz tubes (2 cm in length, 2 mm inside diameter) and quantified using a Mettler microbalance (Mettler–Toledo, Greifensee, Switzerland). The pyrolysis chamber was full of He. The soil samples were heated from ambient to 700 °C at a rate of 20 °C/ms and kept for 15 s. The pyrolyzer was coupled with PerkinElmer Clarus680GC-SQ8MS Systems (PerkinElmer, Santa Clara, CA, USA), and the carrier gas was He. For operation, the temperature program of the capillary column (HP-5, 0.25 mm) of GC was as follows: 3 min at 40 °C, then temperature was increased to 280 °C at a rate of 10 °C/min and kept at 280 °C for 5 min. The injector temperature was 280 °C. The MS indicator was operated in the electron impact mode at electron energy of 70 eV, and the ion source temperature was kept at 250 °C. The pyrolysis products were identified using identifications of the NIST 2014 library and the report by other researchers. Pyrolysis products were quantified by using the surface of two characteristics ion fragments of each product. The relative percentage of squalene compound was calculated according to peak height above baseline. For each sample, the relative peak height of squalene compound was calculated by normalizing results to the largest peak measured in the chromatogram. The percentage of squalene compound reported herein, therefore, is the relative percentage of that compound with respect to the largest peak compounds identified, not the absolute abundance of compounds of squalene in soils.

The soil microbial community was characterized by PLFA analysis. Lipids were extracted from soils by using one-phase chloroform, methanol, and water extractant, then fractionated into neutral lipids, glycolipids, and phospholipids on a silicic acid column. The quantification of PLFAs was performed by GC chromatography (GC Agilent 6890-Agilent Technology, Santa Clara, CA, USA) using a flame ionization detector (FID), split injector, and an HP7673 auto sampler. He as a carrier gas was operated with a flow rate at 0.8 mL min^−1^ and a pressure of 35 psi. The injector and detector temperatures were 250 °C and 300 °C, respectively [30]. PLFAs were designated *X:Y*ω*Z*. *X*: the total number of carbon atoms; *Y*: the number of unsaturated olefinic bond; ω: the end of methyl; *Z*: the location of the keys or cyclopropane chain; a (anteriso) and i (iso): branching chain; 10Me: a methyl group tenth at the end of the pitch molecule carbon atoms; and cy: a cyclopropyl group on the carbon chain [31].

The absolute abundance of PLFAs is expressed as nmol/g dry soil, and the sum of absolute abundances of PLFAs was the microbial biomass [31]. The PLFAs, which were present in <3 samples at very low concentrations, were discarded from analysis. The bacterial PLFAs were estimated as the sum of general bacteria and non-specific bacteria. PLFA biomarkers included i13:0, 14:0, i14:0, i15:0, a15:0, i15:1 G, 16:0, i16:0, 16:1 2OH, 16:1G, 16:1ω5c, 16:1ω9c, i17:0, a17:0, cy17:0, 17:1ω8c, 18:1ω5c, 18:0, and cy19:0ω8c [32,33,34,35,36]. Actinomycete bacteria are represented by 10Me17:0 and 10Me18:0 [37]. Fungal groups included 18:1ω9c [37].

Soil pH was measured electrochemically (Model PHS-3E Meter, Leici Instruments Co. Ltd., Shanghai, China) in H_2_O at a soil: solution ratio of 1:2.5 [38]. Soil organic carbon content was detected by the potassium dichromate sulfuric acid oxidation technique [39]. Total nitrogen was detected by the Kjeldahl method [40]. Total inorganic nitrogen and dissolved organic carbon content were detected by extracting 5 g fresh-weight soil with 25 mL 0.5 mol K_2_SO_4_; then the soil extraction was passed through filter paper, with filtrates then analyzed by an Autosampler (SEAL XY-2 Sampler, Bran & Luebbe, Sydney, Australia) [41].

### 2.4. Data Analysis and Statistics

One-way ANOVA followed by Duncan’s multiple comparisons was employed to test the differences in soil chemistries, including squalene relative abundance, SOC, DOC, TIN, and TN among soils collected from the AM, AS, AMS, ADS, and AD grassland types. The squalene relative abundance and soil chemical/microbial characteristics were subjected to principal component analysis (PCA), based on linear combinations of the original variables on independent orthogonal axes, while the squalene relative abundance and soil chemical/microbial traits were subjected to redundancy analysis (RDA), performed using Canoco 5 (Microcomputer Power, Ithaca, NY, USA, 2012). All statistical analyses were conducted using SPSS 20.0 (IBM, Chicago, IL, USA, 2011) with a significance level of *p* < 0.05. All figures were made by Sigmaplot^®^ Version 10 software (Systat Software Inc., Chicago, IL, USA, 2007).

## 3. Results

### 3.1. Squalene Relative Abundance

**Figure 1:**
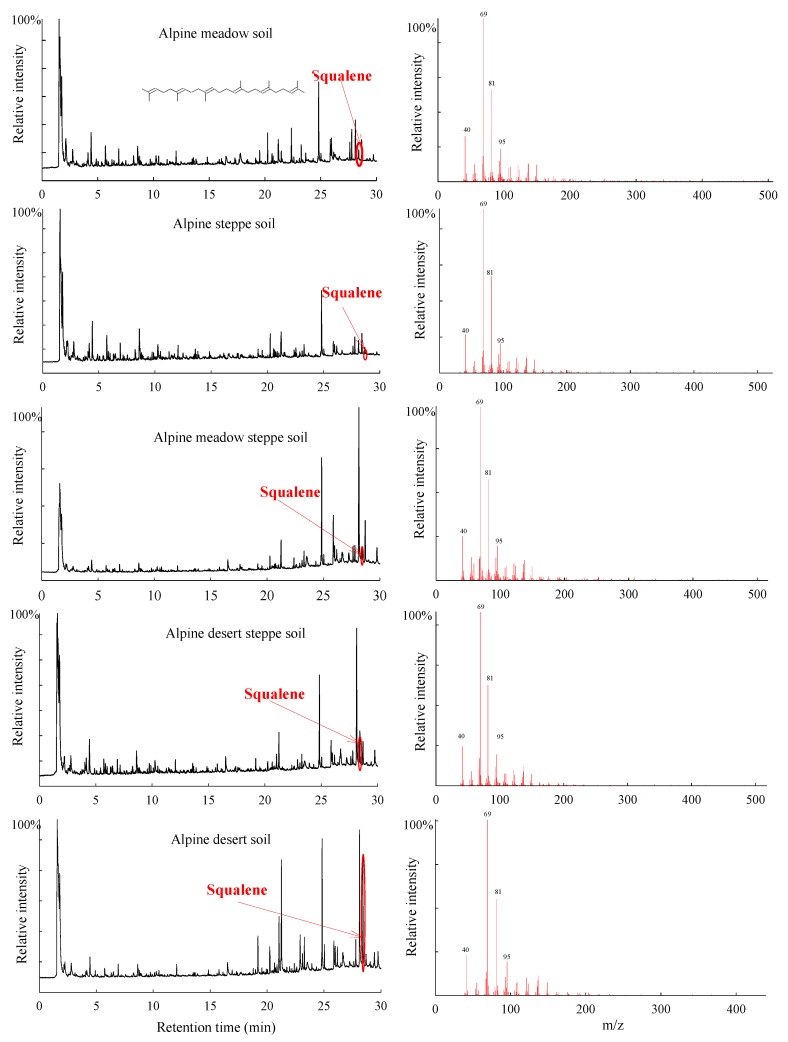
The squalene chromatograms (**left**) and mass spectrums (**right**) obtained from alpine grassland soils by pyrolysis gas chromatography–mass spectrometry (Py-GC/MS) in the Tibetan Plateau. m/z: mass-to-charge ratio.

**Figure 2:**
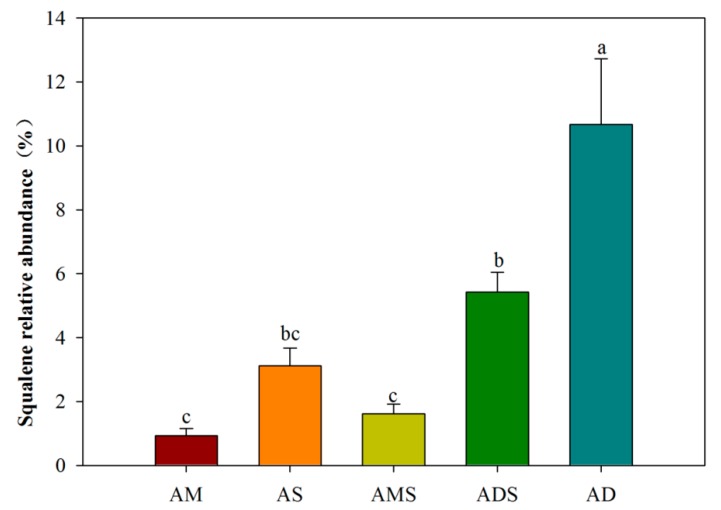
Squalene relative abundance in alpine grassland soils in the Tibetan Plateau. AM: alpine meadow, AS: alpine steppe, AMS: alpine meadow steppe, ADS: alpine desert steppe, AD: alpine desert.

The squalene component was identified from the soils in all five alpine grasslands, including AM, AS, AMS, ADS, and AD in the Tibetan Plateau using Py-GC/MS (Figure 1). There were significant differences in the squalene relative abundance of the soils among five alpine grassland types (Figure 2). The relative abundance of squalene of the soils in AD was the highest, with a value of 10.66 ± 2.07%, and that of the soils in ADS was the second highest, with a value of 5.42 ± 1.38%. The squalene relative abundances of the soils in AM, AS, and AMS were significantly lower than those of the soils in AD, with values of 0.93 ± 0.22%, 3.12 ± 1.23%, 1.61 ± 0.52%, respectively.

### 3.2. Soil Chemical/Microbial Characteristics

Soil pH of the soils in five alpine grasslands was in the range of 7.57–9.52, with the highest value in the AMS soil and the lowest in the AS soil. Significant differences in the soil chemical characteristics were observed among the five types of alpine grassland in northern Tibet (Table 1). The SOC, DOC, TN, and TIN contents were the highest in the AM soil (34.97 ± 2.89 g kg^−1^, 98.39 ± 27.30 mg kg^−1^, 1.18 ± 0.24 g kg^−1^, 39.65 ± 6.68 mg kg^−1^, respectively); these values were 8.02, 4.63, 7.87, and 14.58 times those of the AD soil, respectively, which had the lowest indexes.

The absolute abundance of PLFAs in five grassland type soils showed that the richness order of soil samples was as follows: total PLFA, bacteria, fungi, and actinomycetes (Table 2). The total PLFA values were the highest in the AM soils (23.58 ± 2.76 nmol g soil^−1^), at 2.00, 2.37, 5.30 and 6.45 times those of the AS, AMS, ADS and AD soils. The bacterial PLFAs showed a generally similar pattern to that of total PLFAs. The predominant bacteria were most prevalent in the AM soils (22.84 ± 2.95 nmol g soil^−1^) of these five grassland types, and were least prevalent in AD soil (2.57 ± 0.69 nmol g soil^−1^). The amounts of the fungi and actinomycetes were also the highest in the AM soil, at 3.70 ± 0.54 nmol g soil^−1^, and 1.96 ± 0.27 nmol g soil^−1^, respectively, but the lowest values were found in the ADS soils at 0.96 ± 0.15 nmol g soil^−1^ and 0.37 ± 0.15 nmol g soil^−1^, respectively.

### 3.3. Relationships between Squalene Relative Abundance and Soil Chemical/Microbial Characteristics

**Figure 3:**
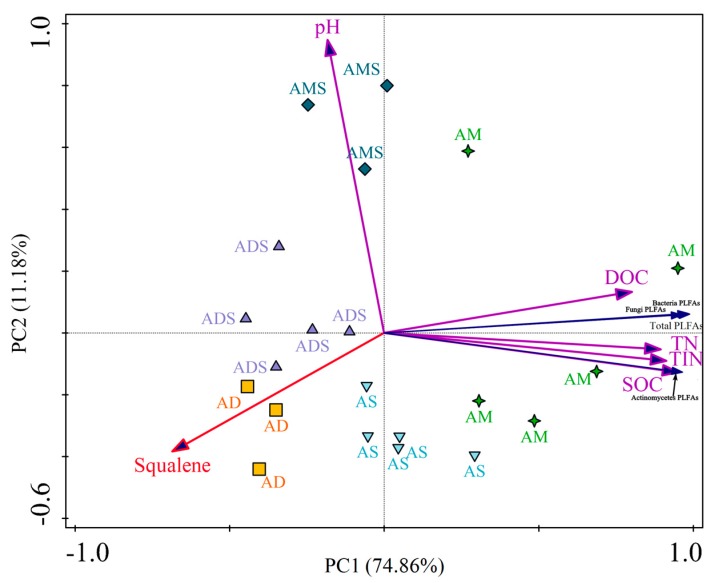
The relationships between squalene relative abundances and soil chemical/microbial characteristics in alpine grassland in the Tibetan Plateau. AM: alpine meadow, AS: alpine steppe, AMS: alpine meadow steppe, ADS: alpine desert steppe, AD: alpine desert, PLFAs: phospholipid fatty acids, SOC: soil organic carbon, DOC: dissolved organic carbon, TN: total nitrogen, TIN: total inorganic nitrogen.

Two principal components were used as tools to distinguish the different grassland ecosystems (AM, AS, AMS, ADS, and AD), considering all properties together (SOC, DOC, TN, TIN, pH, PLFAs, and relative abundance of squalene). The cumulative variation in the distribution of the selected variable was 74.86% and 86.04% for the sum of the principal components PC1 and PC2 in the evaluation performed with soil chemical traits and PLFAs (Figure 3). The analysis of the interaction showed an integrated effect of the grassland ecosystem, soil chemical characteristics, and PLFAs on the relative abundance of soil squalene. For soil chemical characteristics, the relative abundance of squalene had a negative correlation with SOC (*r* = −0.616, *p* < 0.01), DOC (*r* = −0.510, *p* < 0.05), TN (*r* = −0.612, *p* < 0.01), and TIN (*r* = −0.579, *p* < 0.01). Nevertheless, the squalene relative abundance was not in correlation with soil pH. For soil microbial characteristics, the relative abundance of squalene was also significantly negatively correlated with soil PLFA quantity, including soil total PLFA (*r* = −0.642, *p* < 0.01), bacteria PLFAs (*r* = −0.650, *p* < 0.01), fungi PLFAs (*r* = −0.576, *p* < 0.01), and Actinomycete PLFAs (*r* = −0.583, *p* < 0.01).

## 4. Discussion

Squalene is a natural triterpene known to be an important intermediary of cholesterol/phytosterol biosynthesis in animals, plants, humans, and microorganisms. It is used widely in the food, cosmetic, and medicine industries due to its multiple functions [2,4]. Scientists have discovered that when squalene is found in great proportions in some animals and microorganisms, it is likely to be essential to their survival in extreme environments, especially hostile environments free of oxygen. Some animals include sharks, which live in the deep sea with dark, cold, high-pressure, and oxygen-poor conditions [6]. Other examples include moles, which inhabit a damp environment [42], and yaks, which thrive at altitudes of 2000–5000 m above sea level [21]. Squalene is absorbed and distributed to different organs from many biological sources, and is present in varying quantities [5]. In recent years, the bioavailability of squalene has been well established in cell cultures, animal models, and in humans, and further progress has been made concerning on the intracellular transport of this lipophilic molecule. Squalene accumulates in the animal liver and decreases levels of hepatic cholesterol and triglycerides, with these actions being exerted via a complex network of changes in gene expression at both transcriptional and post-transcriptional levels [5].

The Tibetan Plateau, the highest and most extensive highland in the world, is characterized by a harsh environment and fragile ecosystems at high altitude, with strong solar radiation, drought, low temperatures, and poor levels of oxygen [43]. In the present study, the squalene component was identified from five alpine grassland soils in the Tibetan Plateau by using the Py-GC/MS technique. Py-GC/MS served as a valuable analytical technique because pyrolysis products could be separated by gas chromatography and detected by mass spectrometry. The data by Py-GC/MS do not provide insight into the absolute abundance of compounds across samples, an approach that would require multiple internal standards. However, it is an efficient tool for revealing chemical characteristics in the organic matter of soils through semi-quantitative analyses with a comparison of abundance ratios of selected pyrolysis products [44,45,46]. A squalene component was found from all the soils in the five alpine grasslands, with relative abundance ranging from 0.93% to 10.66% in the Tibetan Platea, as shown using the Py-GC/MS technique (Figure 1 and Figure 2). Nevertheless, at present the squalene component has only been found in very few soils in other regions, such as tropical rainforest soils in Indonesia [47], temperate broadleaved forest soils in Belgium [48], Mediterranean forest soil located in Spain [49], and agricultural soil in Canada [50]. For most of the soils, the squalene component was not obtained using the same technique [50,51,52,53,54].

In the Tibetan Plateau, squalene was identified in all the alpine grassland soils and some distribution characteristics are shown in Figure 3. The points were scattered among different alpine grassland types, while the points were concentrated in same grassland type. That is to say, the squalene relative abundances were different among different alpine grassland types, while they were similar in the same alpine grassland type (Figure 3). Comparing the five alpine grassland types, the squalene relative abundance was the highest in alpine desert soils, with a value of 10.66 ± 2.07%, and it was the lowest in alpine meadow soils, with the value 0.93 ± 0.22% (Figure 2). Thus, the relative abundance of squalene relative in alpine desert soils was 11.5 times that of alpine meadow soils. This could be attributed to the different environmental conditions in two alpine grassland types. Alpine deserts are distributed in harsher environments; they are the highest and driest grassland type in China and the world [55]. The average annual temperature ranges from −10 °C to −8 °C, the average annual precipitation from 20.6 mm to 53.8 mm, and the vegetation total cover from 5% to 14% in the alpine desert area. In the alpine meadow area, the average annual temperature is around 0 °C, the average annual precipitation ranges from 450 mm to 600 mm, and the total vegetation cover is from 50% to 90% [55,56,57].

In general, the squalene relative abundance was significantly negatively correlated with soil chemical/microbial characteristics in the Tibetan Plateau (Figure 3). This indicated that the relative abundance of squalene is higher in soils with low microbial quantities and confirmed that squalene is a product of biological adaptation to extreme environments. Soil microbial PLFA quantities in alpine grasslands were positively associated with mean annual temperature, mean annual precipitation, soil organic carbon, and aboveground biomass, and negatively associated with elevation, indicating that the harsh environmental conditions may not benefit the survival of soil microorganisms in the Tibetan Plateau [22,58,59]. Thus, microorganisms may adapt to harsh environmental conditions by increasing the levels of squalene in their bodies. It has been reported that each molecule of squalene could be formed by fusing two molecules of farnesyl diphosphate in microorganisms, and that some special mechanism exists to allow certain microorganisms to independently adapt to extreme environments [3,60]. For instance, prokaryotic hopanoid biosynthesis does not require molecular oxygen as a substrate, and the squalene is directly cyclized by the enzyme squalene-hopene cyclase in hypoxic environments [60]. Squalene has a role in facilitating tighter packing of archaeal lipid mixtures and also influences spatial organization in archaeal membranes of *Halobacterium salinarum*, an extremely halophilic archaeon [61]. Soil organic matter provides energy and the nitrogen elements constituting the nutrients required for the life activity process for microorganisms [62,63]. Therefore, the relationships between squalene abundance and soil chemical characteristics were consistent with its relationships to soil microbes in the Tibetan Plateau (Figure 3).

## 5. Conclusions

Squalene, which is attracting great biological interest due to its beneficial properties and is generally considered to be a product of biological adaptation to extreme environments, was found in all the soils in five alpine grasslands in the Tibetan Plateau using the Py-GC/MS technique. The relative abundance of squalene is higher in soil with low microbial quantities, which in the harsh environmental conditions may not benefit the survival of soil microorganisms in the Tibetan Plateau. This suggests that squalene is possibly a bioactive component for microorganisms in alpine grassland soils to adapt to harsh environmental conditions, especially in oxygen-poor environments. Furthermore, the relative abundance of squalene in alpine grassland soils had a significantly negative correlation with soil chemical/microbial characteristics. Therefore, the harsher the environment, the higher the relative abundance of squalene needed to adapt to this environment in the Tibetan Plateau.

In general, the present study represents preliminary research for squalene in alpine grassland soils; we still do not know which species or populations of microorganisms could biosynthesize squalene, what the mechanism of squalene biosynthesis is in the body of microorganisms, and why and how the extreme environmental conditions stimulate the production of squalene in the soils. In addition, Py-GC/MS is an analytic technique that uses semi-quantitative analyses with a comparison of abundance ratios of selected pyrolysis products. The absolute content of squalene in soils needs to be determined by using an authenticated external standard of squalene. Thus, further in-depth studies concerning squalene distribution, its biosynthesis mechanism, and its relationship with environmental factors are still needed in the Tibetan Plateau.

## Figures and Tables

**Table 1:** The soil chemical characteristics of alpine grasslands in the Tibetan Plateau.

Soil Chemical Indexes	pH	SOC (g kg^−1^)	DOC (mg kg^−1^)	TN (g kg^−1^)	TIN (mg kg^−1^)
AM	8.00 ± 0.15 ^c^	34.97 ± 2.89 ^a^	98.39 ± 27.30 ^a^	1.19 ± 0.54 ^a^	39.65 ± 6.68 ^a^
AS	7.57 ± 0.03 ^d^	17.26 ± 2.48 ^b^	66.21 ± 9.03 ^ab^	0.75 ± 0.32 ^ab^	14.52 ± 2.39 ^b^
AMS	9.52 ± 0.21 ^a^	9.75 ± 5.58 ^bc^	41.91 ± 12.84 ^b^	0.42 ± 0.20 ^bc^	13.58 ± 1.16 ^b^
ADS	8.46 ± 0.29 ^b^	8.74 ± 1.99 ^c^	36.38 ± 6.28 ^b^	0.34 ± 0.12 ^bc^	5.72 ± 2.71 ^b^
AD	8.16 ± 0.11 ^bc^	4.36 ± 0.58 ^c^	21.24 ± 2.73 ^b^	0.15 ± 0.10 ^c^	2.72 ± 1.48 ^b^

AM: alpine meadow, AS: alpine steppe, AMS: alpine meadow steppe, ADS: alpine desert steppe, AD: alpine desert, SOC: soil organic carbon, DOC: dissolved organic carbon, TN: total nitrogen, TIN: total inorganic nitrogen. Values are mean values of soil chemical characteristics ± standard error (S.E.) in alpine grasslands. Values within the same row followed by the same letter are not significantly different at *p* < 0.05.

**Table 2:** The soil microbial composition characteristics of alpine grasslands in the Tibetan Plateau.

PLFAs (nmol g^−1^)	Bacteria	Fungi	Actinomycetes	Total
AM	22.84 ± 2.95 ^a^	3.70 ± 0.54 ^a^	1.96 ± 0.27 ^a^	23.58 ± 2.76 ^a^
AS	11.32 ± 1.43 ^b^	2.03 ± 0.31 ^b^	1.22 ± 0.15 ^b^	11.81 ± 1.41 ^b^
AMS	9.23 ± 1.22 ^bc^	1.86 ± 0.30 ^b^	0.84 ± 0.18 ^bc^	9.96 ± 1.27 ^bc^
ADS	3.72 ± 0.93 ^cd^	0.96 ± 0.15 ^b^	0.36 ± 0.15 ^c^	4.45 ± 0.98 ^cd^
AD	2.57 ± 0.69 ^d^	1.05 ± 0.25 ^b^	0.55 ± 0.11 ^c^	3.66 ± 0.93 ^d^

PLFAs: phospholipid fatty acids, AM: alpine meadow, AS: alpine steppe, AMS: alpine meadow steppe, ADS: alpine desert steppe, AD: alpine desert. Values are mean values of soil microbial PLFA characteristics ± standard error (S.E.) in alpine grasslands. Values within the same row followed by the same letter are not significantly different at *p* < 0.05.
